# Green carbon quantum dots as eco-friendly nano-additives for tribological enhancement of Pongamia oil

**DOI:** 10.1039/d6ra03760g

**Published:** 2026-08-04

**Authors:** Mohammed Ali Khan, Akshatha R. Shetty, Ganesha Aroor, Meghana K. Navada

**Affiliations:** a Manipal Institute of Technology, Manipal Academy of Higher Education Manipal India meghana.navada@manipal.edu

## Abstract

Carbon quantum dots (CQDs) offer various advantages – biodegradability, non-toxicity, lower cost and are used as a replacement for various metal oxide-based nanoparticles. Despite these advantages, the use of CQDs remains limited. This study investigates the synthesis of CQDs using cowpea seeds and their application as nano-additives in bio-based Pongamia oil as a lubricant. The addition of CQDs enhanced the lubricating properties of Pongamia oil. At a concentration of 0.15 wt% CQDs, the nanofluid improved the viscosity and anti-wear properties while reducing the friction compared to the base oil. The enhancement in tribological properties of Pongamia oil after the addition of CQDs was rationalised based on the correlation among viscosity, activation energy, wear, friction, thermal conductivity, flash point, and fire point. The analysis of the wear scar surface revealed lower wear and fewer grooves on the metal surface lubricated with CQD-modified Pongamia oil compared to the base oil. The results offer a simple and effective strategy for using CQDs in base oil as nanoadditives, thereby increasing the tribological properties.

## Abstract 2

Cowpea seed-derived CQDs enhance the tribological and thermal properties of Pongamia oil bio-lubricant by reducing friction and wear. Thus, demonstrating their potential as sustainable nano-additives for green lubrication.

## Graphical abstract

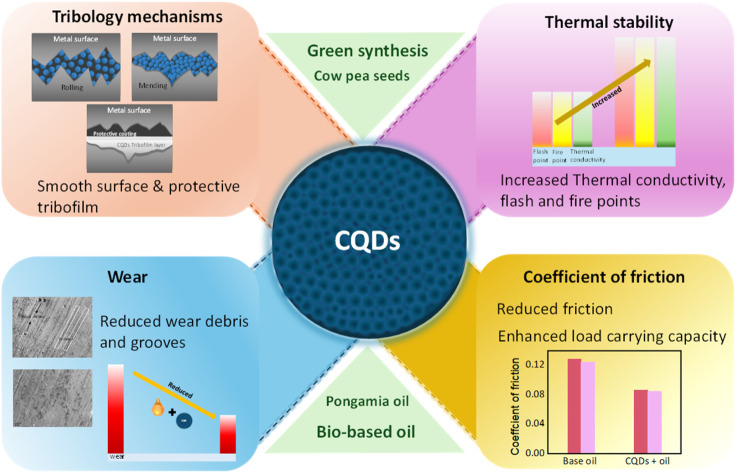

## 1. Introduction

Growing environmental concerns regarding the adverse impacts of conventional mineral oil-based lubricants have driven a shift towards employing bio-lubricants. Although mineral-based oils exhibit higher efficiency of lubrication, their toxicity, poor biodegradability, and non-renewable origin necessitate the^1^ use of eco-friendly lubricants.^2–5^ In this direction, bio-based oils, derived from renewable sources, serve as an alternative sustainable lubricant that minimize the ecological footprint.^6^ In various studies, bio-oils derived from vegetables show superior lubricity compared to mineral-based lubricants, exhibiting promising potential as efficient lubricants for diverse tribological applications.^7–9^ For instance, Saka *et al.* compared the tribological performances of vegetable and mineral-based oils and reported that the lubricity of vegetable oils outperform mineral oils. The vegetable oil exhibited superior oxidative resistance, increased thermal conductivity, and higher contact angles at the solid–liquid interface, as well as a reduced coefficient of friction (COF) compared to mineral oils. These advantages result in improved boundary and mixed lubrication performance, resulting in reduced friction and wear. Consequently, results collectively indicate that vegetable oils are more promising and effective than conventional mineral oils for tribological applications.^10^

Among the various bio lubricants, Pongamia pinnata seed oil (commonly referred to as Pongamia oil) is preferred due to its availability, non-edible nature, and favourable physicochemical properties.^11^ Besides, it exhibits a higher flash point, fire point, physicochemical stability and a lower pour point compared to palm oil.^12,13^ Specifically, the flash point and fire point of Pongamia oil are substantially higher than those of mineral oils, indicating its superior thermal stability and reduced fire hazards at higher operating temperatures. In addition, the selection of oil depends on its oxidative stability, dictated by its fatty acid composition. Polyunsaturated fatty acids such as linoleic acid tend to readily undergo oxidation than monounsaturated fatty acids such as oleic acid.^14^ Owing to its higher oleic acid content, Pongamia oil exhibits greater oxidative stability compared to most other vegetable oils, such as flax and safflower seed oil.^15^

Despite the environmental benefits, vegetable oil-based lubricants inherently face challenges related to degradation that restrict their use in tribological applications. One of the primary issues is the presence of triglycerides in oils that contain bis-allylic hydrogen atoms located near unsaturated fatty acid chains. These atoms are highly vulnerable to free radical attacks and undergo subsequent oxidation. At higher temperatures and oxidative stress, vegetable oils tend to degrade. This includes lipid peroxidation, hyperoxidation, and secondary oxidation. Subsequently, this leads to the formation of lower molecular weight compounds such as ketones, polymers, and aldehydes.^16–18^

To overcome these limitations, the addition of nano-additives to vegetable oil-based lubricants has emerged as an efficient strategy to enhance the tribological and thermal properties.^19–21^ Among various classes of nano additives, metal oxides are extensively utilised due to their stability and ease of synthesis. However, metal oxide nanoparticles (*e.g.* TiO_2_, ZnO) are non-biodegradable and hence can result in toxicity when used in large-scale industries.^22,23^ In this direction, it is essential to develop non-toxic biodegradable carbon-based nanoparticles *via* a green synthesis route with higher efficiency, greater stability, and smaller size.^24^ In this context, carbon-based nanoparticles, such as carbon quantum dots (CQDs), are a revolutionary class of zero-dimensional carbon-based nanomaterials, typically measuring under 10 nm. These CQDs exhibit biocompatibility, lower toxicity, excellent conductivity, eco-friendliness, a higher surface area, fluorescence, and tunable properties, serving as an alternative for tribological applications.^25^ CQDs can be synthesised *via* bottom-up approaches such as the hydrothermal method using renewable biomass precursors (plants, agricultural waste). For instance, Yadav *et al.* synthesised CQDs using a hydrothermal process by taking *Azadirachta indica* as a plant precursor, which was further used for the detection of chemicals in fresh fruits.^26^

Table 1 lists various green precursors used to synthesize CQDs and other carbon based nano-additives. Though several biomass precursors are explored, the use of *Vigna unguiculata* (cowpea) seeds as a carbon source for the synthesis of CQDs has not been reported. Furthermore, no studies were identified that investigate the incorporation of cowpea-derived CQDs into Pongamia oil for tribological applications. This work presents the novel synthesis of CQDs from *Vigna unguiculata* and the use of CQDs as nano-additives in Pongamia oil for tribological applications. The synthesised CQDs were evaluated for their structural and morphological properties and their role as nano-additives on the thermo-rheological properties of Pongamia oil.

**Table 1:** Comparative table on the previous studies involving biomass-derived lubricant additives

Additive	Synthesis/source	Concentration	Base oil	Performance	References
Sugar cane bagasse activated carbon	Steam/acid activated carbon sugar cane bagasse (Biochar)	Bulk	Dry film	COF ∼ 0.08	Molza *et al.*^27^
SCQDs and ACQDs	Safflower and *Angelica sinensis*	5 mg mL^−1^	Water	COF: SCQDs-0.13, ACQDs-0.12	Qiang *et al.*^28^
CQDs	Pyrolysis/onion peel	2% volume	Araldite LY556 epoxy resin	COF-0.22	S. P. M. *et al.*^29^
CDs	Solvothermal/ginkgo leaves	0.20 wt%	PEG-200	COF-0.137	Mou *et al.*^30^
Porous activated carbon	Carbonization/rice husk	10 wt%	Epoxy resin	COF-0.435	Yadav *et al.*^31^

## 2. Materials and methods

### 2.1. Synthesis of CQDs

The CQDs were synthesised *via* a modified one-step, facile hydrothermal process using cowpea (*Vigna unguiculata*, purchased from the commercial market) seeds as a carbon-rich precursor. The seeds were thoroughly washed and sun-dried for 4 days. The dried seeds were manually ground using a mortar and pestle. Subsequently, 6 g of precursor was dispersed in 60 mL of deionised water (neutral pH) and ultrasonically treated for 20 min to ensure uniform mixing. The resulting homogeneous suspension was transferred to a 100 mL Teflon-lined stainless steel autoclave. The reaction mixture was heated at 200 °C for 22 h until the solution changed from transparent to pale yellow, indicating the formation of CQDs. The reaction mixture was cooled to ambient temperature and purified by centrifuging at 12 000 RPM for 20 min to remove larger particulates. The supernatant was again purified using a dialysis membrane (Himedia, Dialysis Membrane-150; molecular weight cut-off (MWCO) of 8000 Da) for 72 h, with water changes every 12 h. A subsequent centrifugation step (12 000 RPM) yielded pure CQDs. Finally, the purified CQDs were freeze-dried at approximately −80 °C to obtain CQD nanoparticles.

### 2.2. Preparation of Pongamia oil CQDs nano lubricants

Pongamia oil was used as a lubricant and was procured from a local vendor in Udupi District, Karnataka, India. For each concentration, 250 mL of Pongamia oil was utilized. CQDs modified Pongamia oils were prepared by dispersing 0.05, 0.1, 0.15 wt% CQDs (based on oil mass) into the oil. The homogeneity of the CQD nanoparticles was ensured by ultrasonication.

### 2.3. Characterization tools

The optical absorption of CQDs was measured using a UV/Vis/NIR spectrometer (Shimadzu UV-3600). Functional group identification was performed by Fourier Transform Infrared (FTIR) spectroscopy using a Shimadzu 8400-FTIR spectrometer. Crystallographic analysis was conducted by X-ray diffraction (XRD) using a Rigaku Ultima IV equipped with Cu-Kα radiation (*λ* = 1.54 Å) as a radiation source. The interplanar *d*-spacing was calculated using Bragg's law.^32^1
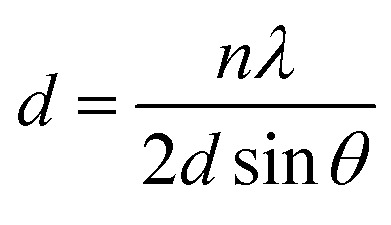
In which *n* is the order of reflection, *λ* is the X-ray wavelength, *d* is the interplanar spacing and *θ* is the Bragg's angle.

Scanning electron microscopy (SEM) and energy-dispersive spectroscopy (EDS) were performed using an EVO MA18 with Oxford EDS(X-act) under liquid nitrogen-free conditions, respectively. High-resolution transmission electron microscopy (HR-TEM) imaging was performed using JEOL JEM-F200 operated at 200 kV. Zeta potential measurements were obtained using the Horiba nanoparticle analyser with dispersion medium viscosity of 0.894 mPa s and a conductivity of 0.523 mS cm^−1^.

### 2.4. Tribology instrumentation

Viscosity was measured using an Anton Paar MCR 92 rheometer, operated with Anton Paar RheoCompass 1.31 software. The software was programmed to measure shear stress (PaS) at the interval ranges of 0–100 s^−1^, and the instrument was calibrated for each experiment. The experiment was conducted by taking 10 mL of the sample into the base fixed plate, and it was performed at temperatures of 10, 20, 30, 40, 50, 60, 70, and 80 °C. After each experiment, the base plate was cleaned with ethanol to remove any sample residue, and each experiment was conducted three times to ensure repeatability. Viscosity was measured using the Herschel–Bulkley model with rotating shear mode. The flow activation energy was calculated using the Arrhenius equation, as previously described by Kim *et al.*^33^

2 *η* = *A*e^*E*_a_/*RT*^

3
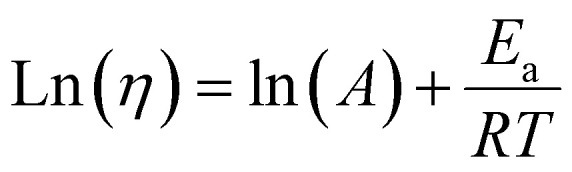
where *η* is the dynamic viscosity. The flow activation energy, *E*_a_ (J mol^−1^) is calculated by plotting the natural logarithm of viscosity [ln(*η*)] against the reciprocal of temperature (K). This results in a linear curve with a slope of (*E*_a_/*R*), where *R* is the universal gas constant (8.314 J mol^−1^ K^−1^).^34^ This method enables the measurement of the energetic threshold associated with viscous flow.

The shear stress *vs.* shear rate data were fitted, and the flow behaviour index of the Pongamia oil and CQDs modified Pongamia oils was calculated using the Herschel–Bulkley model.

4 *σ* = *σ*_*y*_ + *k

<svg xmlns="http://www.w3.org/2000/svg" version="1.0" width="10.615385pt" height="16.000000pt" viewBox="0 0 10.615385 16.000000" preserveAspectRatio="xMidYMid meet"><metadata>
Created by potrace 1.16, written by Peter Selinger 2001-2019
</metadata><g transform="translate(1.000000,15.000000) scale(0.013462,-0.013462)" fill="currentColor" stroke="none"><path d="M320 960 l0 -80 80 0 80 0 0 80 0 80 -80 0 -80 0 0 -80z M160 760 l0 -40 -40 0 -40 0 0 -40 0 -40 40 0 40 0 0 40 0 40 40 0 40 0 0 -280 0 -280 -40 0 -40 0 0 -80 0 -80 40 0 40 0 0 80 0 80 40 0 40 0 0 80 0 80 40 0 40 0 0 40 0 40 40 0 40 0 0 80 0 80 40 0 40 0 0 120 0 120 -40 0 -40 0 0 -120 0 -120 -40 0 -40 0 0 -80 0 -80 -40 0 -40 0 0 200 0 200 -80 0 -80 0 0 -40z"/></g></svg>


*^*n*^

where, *σ* is the shear stress (Pa), *σ*_*y*_ is the yield stress (Pa), *k* is the consistency index (Pa s^*n*^), *γ̇* is the shear rate (s^−1^), and *n* is the flow behavior index.

The lubrication characteristics were studied using the OFFITE lubricity tester. This measures the resistance of the fluid layer between two closely spaced metallic surfaces. It employs flooded lubrication in accordance with ASTM standards. It follows the principles of a block-on-ring lubricated condition to measure the COF. The experiment was conducted using 200 mL of sample. The COF was measured at 60, 80, 100, 120, and 140 RPM for 20 min. The applied loads were 66.66, 100, and 133.33 N, and each experiment was conducted three times to ensure consistency and repeatability. The wear property was studied using a TR 20 NEO Wear and Friction Monitor (pin-on-disk tribometer) following the ASTM G99 standards. The experiment was conducted using 15 mL of sample at room temperature. The brass material (C28000) was used as a pin, as it is widely used in friction-intensive applications^35^ such as brakes and piston cylinders and has good mechanical and corrosion resistance, with a wear track diameter (WTD) of 60 mm. Sliding distances for 200 and 400 RPM were 1130.4 meters and 2260.8 meters, respectively. The loads were 20 N and 40 N, and the speeds were 200 and 400 RPM. Before each trial, the rotating disc was cleaned using ethanol to remove any residue. Thermal conductivity of the samples was determined using a KD-2 Pro Tempos Thermal Properties Analyzer with a range of 0.02–2.00 W m^−1^ K^−1^. The KS-3 sensor was used for the analysis, the sensor was immersed in the 20 mL samples taken in a sample container. To ensure accuracy, the sensor probe was calibrated before each trial. The test was performed for 15 min after immersing the sensor probe at 28 °C (room temperature). The experiment was conducted three times for repeatability.

## 3. Results and discussion

### 3.1. Analysis of Pongamia oil as a bio-lubricant

The physicochemical stability of the oil depends on its free fatty acids, the degree of unsaturation in triglycerides and the concentration of hydroperoxides. To determine these, acid, iodine, saponification and peroxide tests are essential. Hence, these tests are performed (Table 2) to evaluate the extent of oxidation, hydrolytic degradation, the composition of oil, its suitability and expected performance in tribological applications.

**Table 2:** The physicochemical tests of Pongamia oil

Tests	Values
Acid value	2.650 mg KOH per g
Iodine value	76.146 g I_2_ per 100 g
Saponification value	186 mg KOH per g
Peroxide value	4.0 meq. O_2_ per kg
Density	916.64 kg m^−3^ at 31 °C

### 3.2. Structural and morphological characterisation of CQDs

**Fig. 1:**
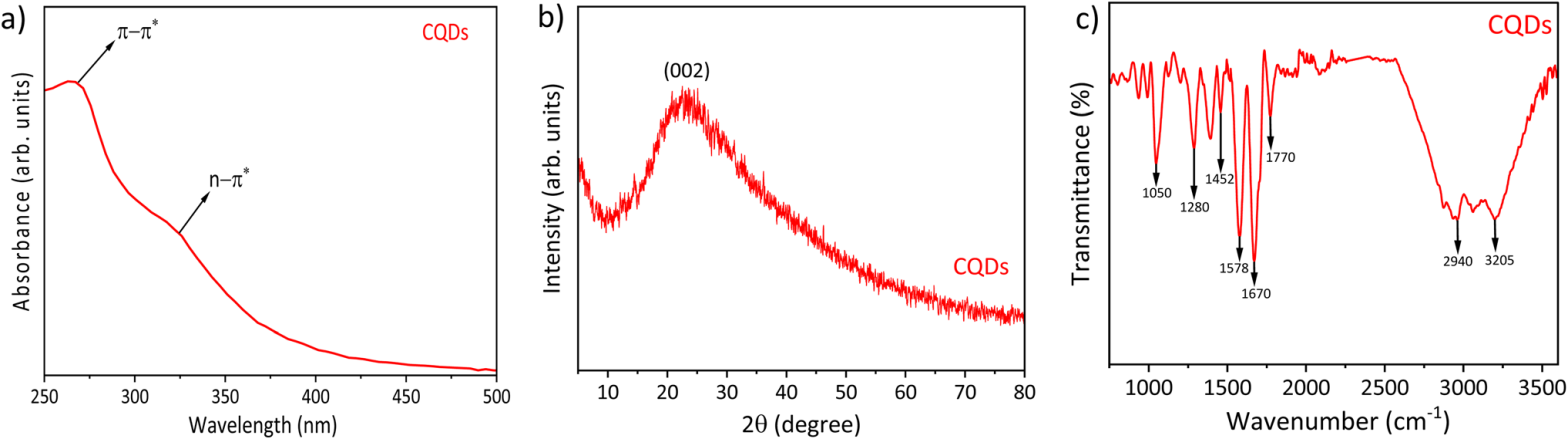
(a) Absorption spectra, (b) XRD pattern and (c) FTIR of the synthesized CQDs.

To confirm the synthesis of CQDs, optical absorption and XRD analysis were performed. In Fig. 1a,CQDs exhibited two characteristic absorption peaks at 268 nm and 306 nm. The absorption peak originating at 268 nm is attributed to π–π^*^ transitions of the graphitic core by aromatic sp^2^ hybridisation of conjugated C=C double bonds. In contrast, the peak at 306 nm indicates n–π^*^ transitions of carbonyl C=O moieties, corresponding to oxygen-containing functional groups located on the surface of CQDs.

The XRD pattern of the synthesized CQDS, depicted in Fig. 1b, shows a broad peak at 22.3°. This broad peak reveals the amorphous nature and disordered carbon of CQDs. The *d*_002_ = 0.34 nm (eqn (1)), which is higher than the interplanar distance of graphite (*d*_002_ = 0.33 nm), reveals the graphitic sp^2^ hybridization of the carbon core of CQDs. The increased *d* spacing and broadness of the peaks reveal higher structural disorder due to surface functionalization.^36^ Hence, to determine the functional groups present on the surface of the CQDs, FTIR analysis was performed.

The FTIR spectrum in Fig. 1c revealed broad peaks at 3205 cm^−1^ and 2940 cm^−1^, corresponding to the overlapping asymmetrical and symmetrical stretching vibrations of N–H, O–H groups, and C–H groups, respectively. A distinct band at 1770 cm^−1^ is assigned to stretching vibrations of carbonyl (C=O) groups. The peaks at 1670 cm^−1^ correspond to N–H bending vibrations, while the peak at 1578 cm^−1^ is associated with C=C stretching vibrations. The C–H bending and C–O–C stretching peaks are ascribed at 1452 cm^−1^ and 1280 cm^−1^, respectively. The presence of C–O stretching vibrations is confirmed by the peak at 1050 cm^−1^.^37^ Overall, FTIR analysis suggests the presence of amino, hydroxyl, and carboxylic/carbonyl moieties, confirming the highly functionalized surface of the CQDs, which is consistent with XRD data.

**Fig. 2:**
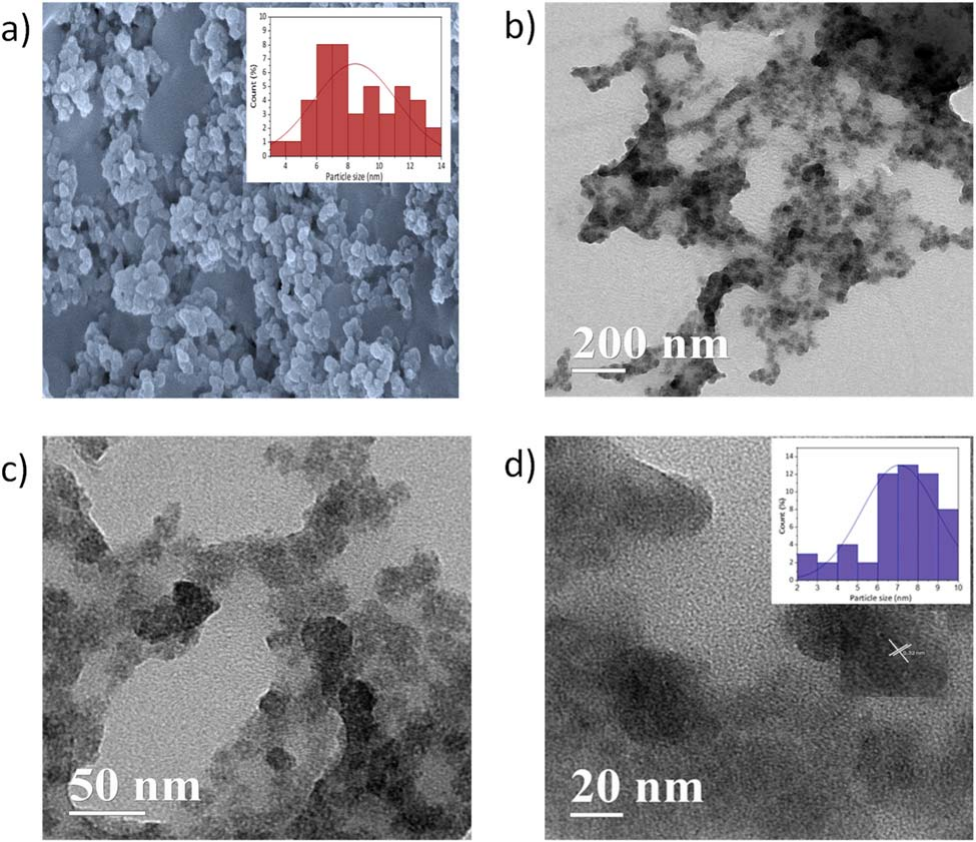
(a) The SEM image with particle size histogram of synthesized CQDs. (b) and (c) HR-TEM images of the synthesized CQDs with 200 nm and 50 nm respectively. (d) HR-TEM image of synthesized CQDs showing particle size histogram and interplanar indices.

To further analyze the structure and morphology of the synthesized CQDs, SEM analysis was performed. Fig. 2a exhibits that the synthesized CQDs are spherical-shaped nanoparticles. The average particle size of the CQDs, determined from the SEM images, is 8.46 ± 1.3 nm. The HR-TEM images in Fig. 2b of synthesized CQDs revealed a morphology that is consistent with SEM analysis. In Fig. 2c, it can be observed that CQDs are well dispersed without agglomeration, indicating good colloidal stability, corroborated by zeta potential. The particle size distribution depicted in Fig. 2d exhibits the size distribution of the synthesized CQDs, which range from 2–10 nm with an average particle size of 7.06 ± 1.8 nm, corroborating the nanoscale dimensions observed *via* SEM and HR-TEM.

Additionally, Fig. 2d exhibits an interplanar spacing of 0.32 nm, which corresponds to the (002) plane of graphitic carbon, suggesting the presence of a crystalline graphitic core within the CQDs.

The zeta potential analysis was conducted to investigate the stability of the synthesized CQDs. Fig. S1 indicates a zeta potential of −29.9 mV, exhibiting good colloidal stability. The net negatively charged rich surface depicts a higher amount of oxygen-containing functional groups (C=O, O–H), in agreement with the FTIR and XRD analyses.

**Fig. 3:**
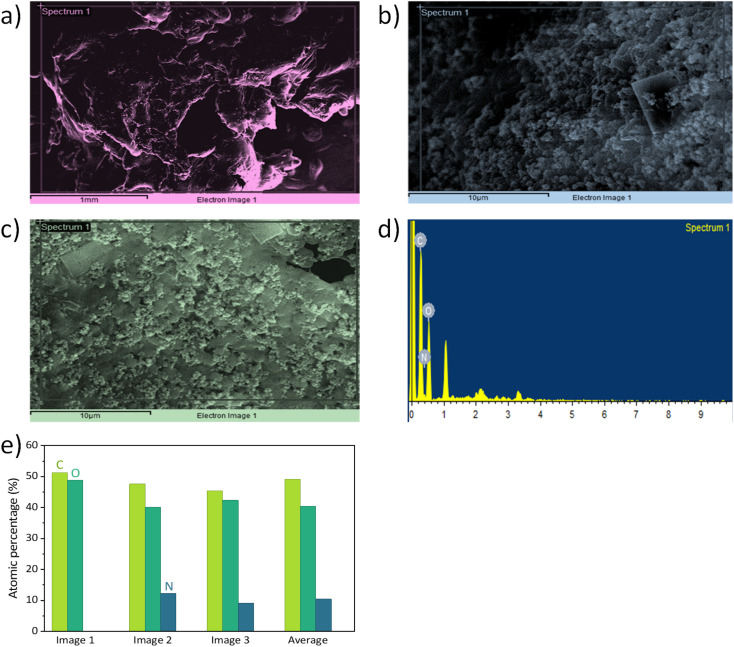
(a) EDS images of the synthesized CQDs at 1 mm magnification. (b) and (c) EDS images of the synthesized CQDs at 10 mm magnification. (d) Elemental composition of the synthesized CQDs. (e) The atomic percentages of synthesized CQDs deduced from EDS measurements at three different sites.

The EDS analysis was performed to understand the elemental composition present in synthesized CQDs (Fig. 3a). The elemental spectrum in Fig. 3d confirms the presence of carbon, oxygen and nitrogen. The average atomic distribution in Fig. 3d reveals carbon is the most abundant element with 49.15%, followed by O at 40.39% and N at 10.49%.

### 3.3. Dispersion stability of CQDs in Pongamia oil

**Fig. 4:**
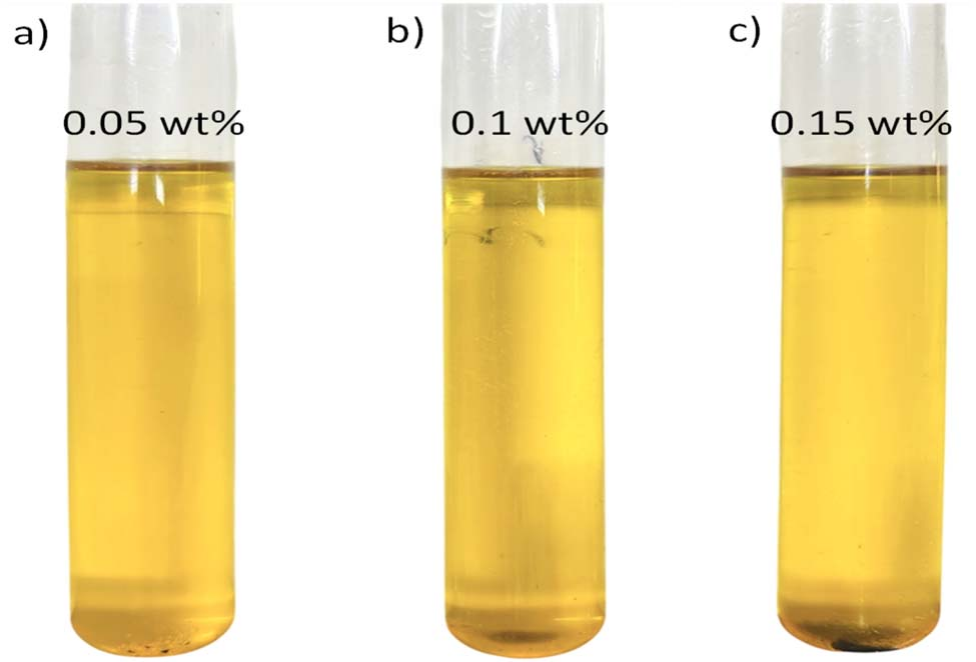
Photographs of Pongamia oil for different concentrations of (a) 0.05 wt%, (b) 0.1 wt% and (c) 0.15 wt% of CQDs.

The dispersion stability analysis was performed by taking CQDs as nanoadditives and Pongamia oil as base oil. As depicted in Fig. 4, the CQDs exhibit a good dispersion stability in Pongamia oil. After 1 month, the Pongamia lubricant with 0.05 wt% CQDs exhibited transparent and clear fluid without much sedimentation. However, 0.15 wt% exhibits slight precipitation but is still homogeneous and opaque in nature. The zeta potential analysis was conducted to investigate the stability of the 0.15% CQDs modified Pongamia oil. Fig. S2 indicates a zeta potential of −27.0 mV, exhibiting good colloidal stability. This dispersion stability enables CQDs modified Pongamia oil as a promising lubricant for tribological applications.

### 3.4. Thermo-rheological behaviour

#### 3.4.1. Viscosity and flow activation energy

**Fig. 5:**
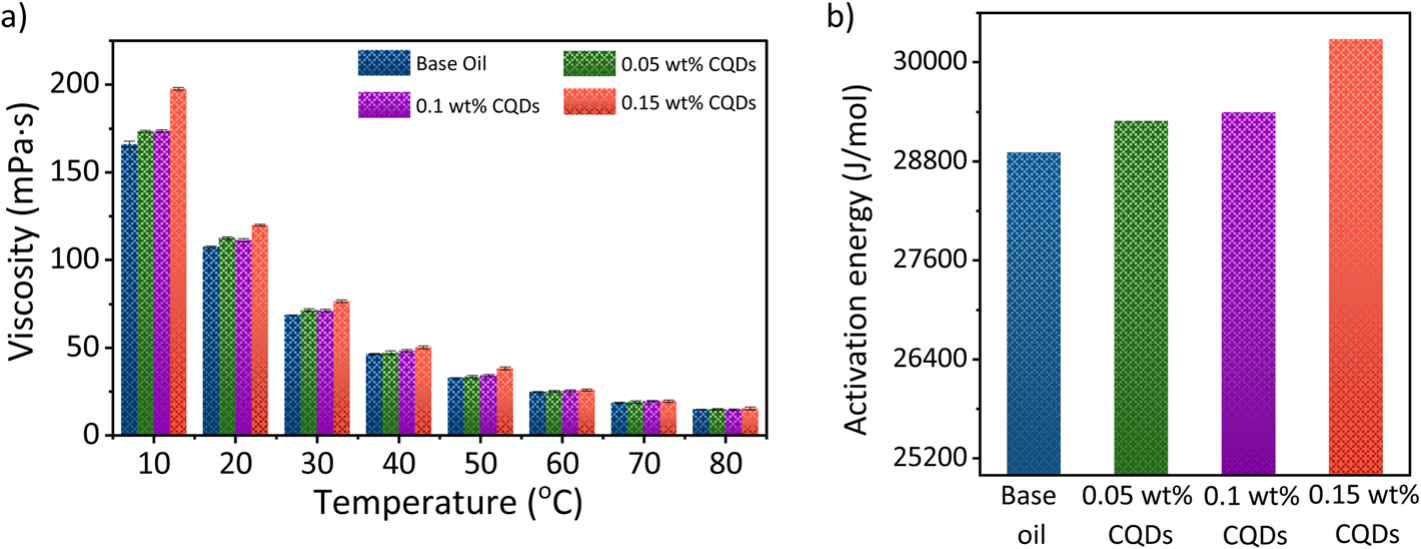
(a) A graph of temperature-dependent dynamic viscosity for base Pongamia oil and CQDs modified Pongamia oils (0.05–0.15 wt%). (b) A bar graph of calculated activation energy for base Pongamia oil and CQDs modified Pongamia oils (0.05–0.15 wt%).

Fig. 5a exhibits the viscosity of the Pongamia oil and CQDs modified Pongamia oil, which was evaluated at different temperatures (10–80 °C). There is an inverse correlation between temperature and viscosity of nanofluids, indicating Arrhenius behavior. As the temperature increases, the viscosity of the lubricant decreases, a characteristic of liquid lubricants. This results from molecular mobility and weak intermolecular interactions. Lower viscosity leads to the liquid moving away, causing the closely moving parts to fail, leading to higher breakage and wear.

In Fig. 5a, the base Pongamia oil exhibits relatively the lowest viscosity across the entire thermal range, while the addition of CQDs substantially enhanced the viscosity. For instance, at 10 °C, the viscosity increased from 167 mPa s for base Pongamia oil to 197 mPa s for the 0.15% CQDs modified Pongamia oil. This enhancement is due to the hydrodynamic effect of the interaction between CQDs suspended in the oil. Furthermore, the oxygen-containing surface functionalisation of CQDs facilitates hydrogen bonding and physicochemical interactions with polar components of the oil. These interactions result in internal resistance to flow, restricting molecular mobility and leading to higher viscosities at lower temperatures.

To evaluate the minimum amount of energetic threshold needed for the molecular movements in the fluid, activation energy was deduced using the Arrhenius model (eqn (2)) (Table S1). At higher temperatures, lubricants requiring lower activation energies readily overcome the energy barrier, resulting in increased sensitivity to thermal fluctuations and thereby a reduction in viscosity. Conversely, lubricants with higher activation energies are thermally stable at higher temperatures, with higher viscosity and increased energetic requirement for molecular movement, thereby maintaining their viscosity across the temperature range. The difference in activation energies for lubricants plays a crucial role in determining the rheological and temperature-dependent properties of lubricants.

In Fig. 5b, the flow activation energies of base Pongamia oil and CQDs modified Pongamia oils (0.05–0.15 wt%) exhibit that the Pongamia oil has a lower activation energy (28 830 J mol^−1^) compared to CQDs modified Pongamia oils Table S1. Therefore, the reduced activation energy is related to the lower viscosity of the base Pongamia oil throughout the temperature range.^38^ As a result, the base Pongamia oil is less effective as a lubricant; its reduced film strength under a higher load makes the protective boundary layer susceptible to breakage, thereby increasing the surface wear and breakage of machinery.

The increased flow activation energy in CQDs modified Pongamia oils denotes an increased thermodynamic barrier to molecular motion by the CQDs. This supports Pongamia oil as a suitable lubricant for the formation of a stable tribo-film.

#### 3.4.2. Flow behaviour

**Fig. 6:**
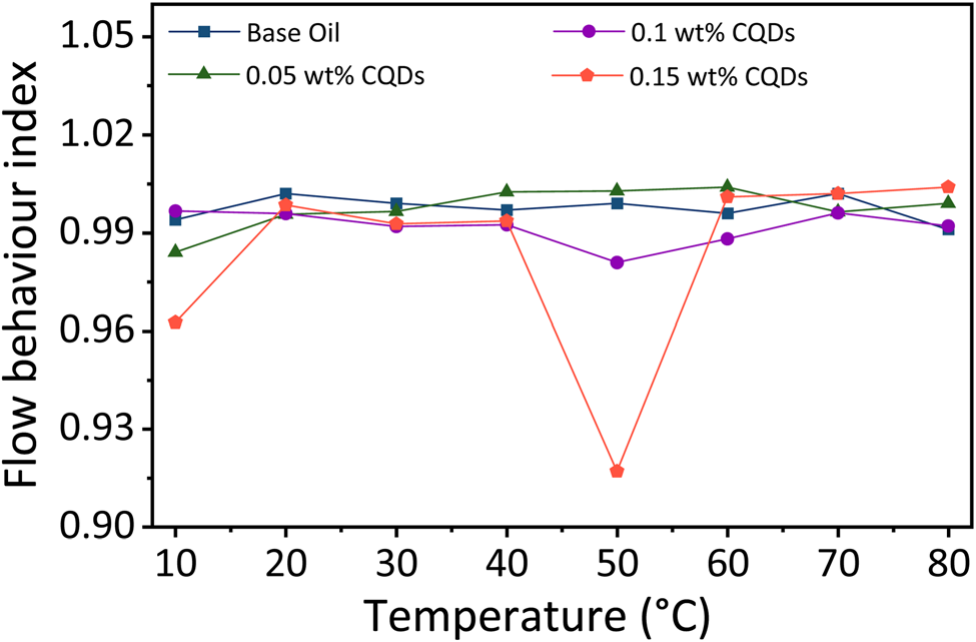
Flow behavior index of the base Pongamia oil and CQDs-modified Pongamia oil nanofluids (0.05–0.15 wt%).

Fig. S3 represents the flow curves of base Pongamia oil and CQDs modified Pongamia oil as a function of temperature. Both fluids revealed Newtonian fluid characteristics, confirmed by shear stress-shear rate curves intersecting the origin. Thereby, ruling out the shear thickening and shear thinning characteristics of fluid and confirming the absence of non-Newtonian behavior. The slope of the curves increases across the temperature range while maintaining the Newtonian characteristics of the fluid. These curves were also fitted to Herschel–Buckley's model (eqn (4)) observed in Fig. 6 and Table S2. The flow behavior was further analysed to understand the fluid's dependence of shear stress on shear rate. Fluids with a flow index *n* < 1 exhibit shear thinning behavior, those with *n* = 1 then exhibit Newtonian flow, and fluids with *n* > 1 exhibit shear thickening behavior.

Base Pongamia oil in Fig. 6 exhibited a flow behavior index of ∼1.0 across the temperature range, indicating Newtonian behavior. Upon the addition of 0.05 wt% of CQDs, there is no notable change observed, indicating no shear thickening or thinning at this concentration. At higher concentrations of CQDs (0.1–0.15%), there is a notable dip in flow behavior at 50 °C, indicating an enhanced shear-thinning behavior at this temperature. The sudden reduction is attributed to the formation of flocculated microstructures at higher concentrations of CQDs. The relative dominance of interparticle force over particle fluid stabilisation results in shear thinning behavior. The flow trend recovers at higher temperatures due to increased Brownian motion and reduced interparticle interaction. Hence, it leads to the weakening of these networks, thereby restoring Newtonian flow behavior.^39–41^

### 3.5. Wear analysis

The wear study was conducted to determine the mass loss from the brass material due to sliding friction between the pin and disk. Higher mass loss due to friction suggests poor lubrication, leading to surface damage and shorter component life. On the contrary, lower mass loss suggests efficient lubrication due to stable tribofilm formation, leading to relatively less material loss with effective surface protection and longer component lifespan.^42,43^

**Fig. 7:**
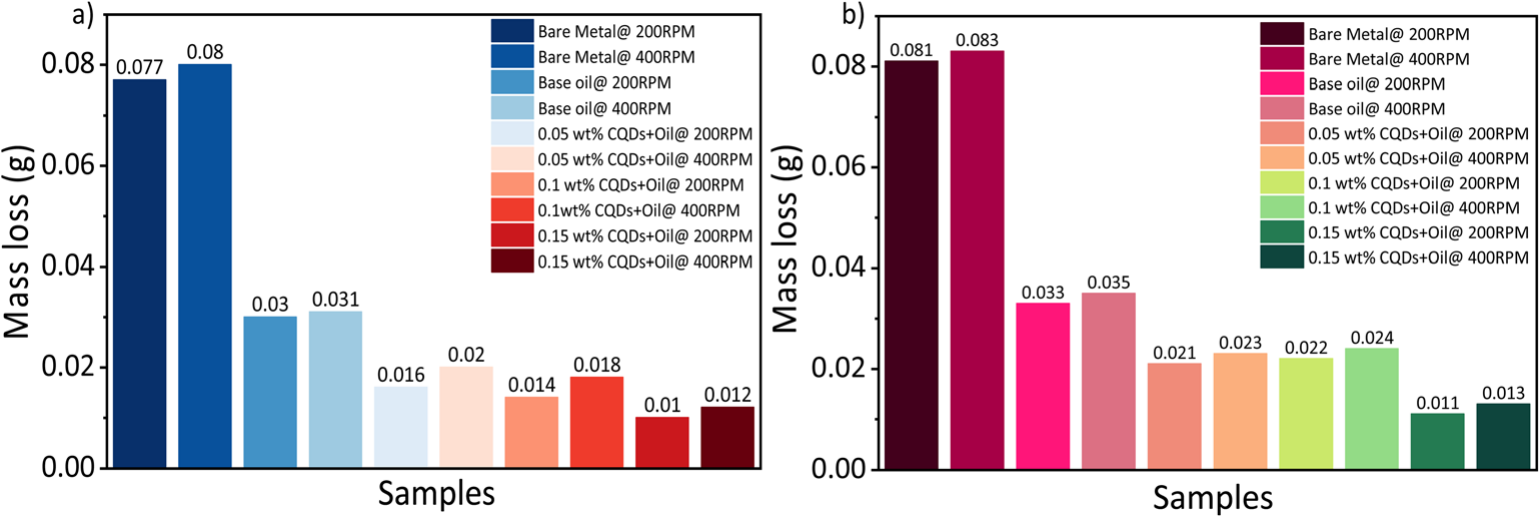
Mass loss of the samples under wear analysis. (a) Bare metal, base Pongamia oil and CQDs modified Pongamia oils (0.05–0.15 wt%) evaluated at 20 N and rotational speeds 200 400 RPM. (b) Corresponding, mass loss at 40 N and rotational speeds of 200 400 RPMs.

At a load of 20 N, the bare metal exhibited relatively highest mass loss as observed in Fig. 7a, indicating higher wear of material due to direct metal contact in the absence of lubrication. The addition of base Pongamia oil decreased the mass loss to 0.030 and 0.031 g at 200 and 400 RPM, respectively. The addition of CQDs further decreased the mass loss by ∼50% compared to base Pongamia oil. Among all the CQDs nanofluids, the 0.15 wt% CQDs exhibited the lowest mass loss (0.010 and 0.012 g), corresponding to a relative reduction of ∼86% compared to bare metal conditions. Thereby, demonstrating a anti-wear property of CQDs modified Pongamia oils.

As shown in Fig. 7b, all the samples exhibited relatively higher mass loss at higher loads of 40 N due to the increased contact stresses, leading to the thinning of the fluid film. The bare metal exhibited the highest mass loss, consistent with the trend observed at 20 N. Among all the CQDs modified Pongamia oils, 0.15 wt% CQDs exhibited a relative reduction of ∼85% compared to bare metal conditions. Thereby confirming its effective anti-wear performance by the formation of a possible protective layer.

**Fig. 8:**
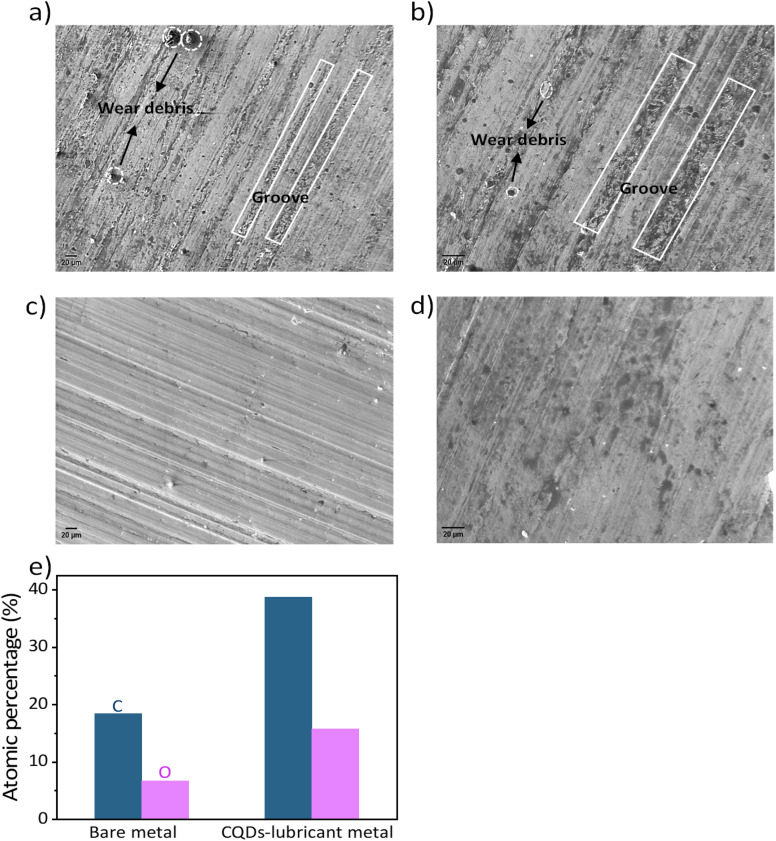
SEM images of wear scar surfaces (a) and (b) bare metal without the addition of lubricant. (c) and (d) Metal after the addition of CQDs modified Pongamia oil. (e) EDS of atomic percentage of bare metal and CQDs lubricant metal.

To understand the role of CQDs as an additive in the lubricating mechanism, the wear surfaces of the bare metal and the CQD modified nanofluid were analyzed using SEM. The SEM images in Fig. 8a and b exhibited deep grooves and higher wear debris on the wear scar surface of the bare brass metal without any lubrication. Fig. 8c and d exhibited a relatively smoother surface with fewer grooves, and reduced wear debris on the wear scar of the metal surface lubricated with CQDs-modified Pongamia oil. The nanoscale size of CQDs and the presence of abundant oxygen-containing functional groups on the surface of the CQDs contribute to load absorption, thereby reducing wear of the metal. The EDS analysis (Fig. 8e and S4) of the worn surface lubricated with CQD-modified Pongamia oil revealed a higher relative content of carbon and oxygen compared to the base oil, suggesting the deposition of carbonaceous species on the worn surface. This suggests the possible development of a carbon-rich protective layer during sliding.

### 3.6. Coefficient of friction

The COF analysis was conducted to determine the effectiveness and lubricity of the lubricant for the reduction of friction between two surfaces.

**Fig. 9:**
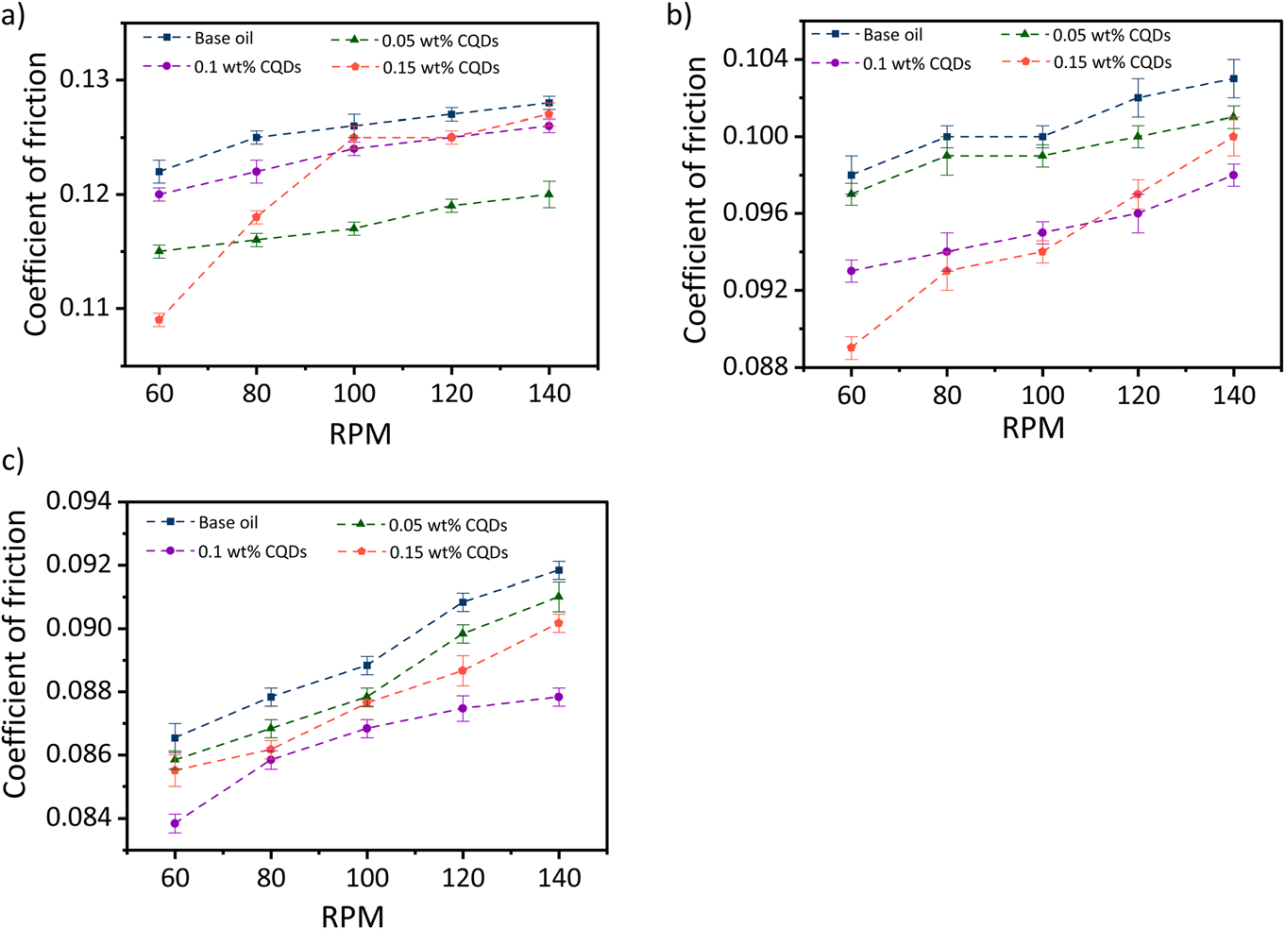
(a) COF of base Pongamia oil and CQDs-modified Pongamia oil nanofluids (0.05–0.15 wt%) at 66.66 N load. (b) COF of base Pongamia oil and CQDs-modified Pongamia oil nanofluids (0.05–0.15 wt%) at 100 N load. (c) COF of base Pongamia oil and CQDs-modified Pongamia oil nanofluids (0.05–0.15 wt%) at 133.33 N loads.

Base Pongamia oil exhibited the highest COF across all the loads and RPM. The incorporation of CQDs (0.05–0.15 wt%) relatively reduced the COF by 34% compared to base Pongamia oil. As depicted in Fig. 9a, the base Pongamia oil exhibited the highest COF (0.128) at 66.66 N. Addition of CQDs reduced the COF, with 0.15 wt% CQDs modified Pongamia oil exhibiting 0.107 at 60 RPM. At higher RPM, 0.05 wt% CQDs exhibited the lowest COF relative to base Pongamia oil. This reduction in COF could be attributed to possible more stable protective layer formation during sliding.

As shown in Fig. 9b, at 100 N, the COF values for all the samples were slightly reduced compared to 66.66 N. The base Pongamia oil exhibited the highest COF (0.103), whereas 0.15 wt% CQDs modified Pongamia oil exhibited a reduction of 13.6% for major RPM. At higher RPM, 0.1 wt% CQDs modified Pongamia oil exhibited the lowest COF(0.098), indicating the addition of CQDs enhances load-bearing capacity and distributes the contact stresses uniformly across the two closely moving metal surfaces.

At the maximum load of 133.33 N (Fig. 9c), the COF values decreased compared to 66.66 N. Base Pongamia exhibited a COF value of 0.092. At 0.15 wt% CQDs, the COF is slightly increased due to particle interaction under sliding conditions for higher loads. 0.1 wt% CQDs modified Pongamia oil consistently exhibited the lowest COF (0.084) across all the RPMs, indicating the optimum concentration of CQDs for 133.33 N. It corresponds to ∼9% relative reduction in COF compared to base Pongamia oil, indicating a plausible hydrodynamic lubricating regime and thereby confirming the formation of a possible protective layer. The COF obtained in the present study was compared with previously reported carbon-based and nanomaterial lubricant additives under different testing conditions, as summarized in Table 3.

**Table 3:** A comparative literature table summarizing COF, additive concentration, base oil, and testing conditions

Additive type	Concentration	Base oil	Test conditions	COF	References
CQDs and Ni-CQDs	CQDs-2 wt%	Polyethylene glycol-200 (PEG-200)	Ball on disk, 100 N	CQDs ∼ 0.18	Tu *et al.*^44^
	Ni-CQDs-2 wt%			Ni-CQDs ∼ 0.14	
CQDs	0.05 wt%	Polyalphaolefin (PAO)	Four ball tester, 15 N	0.076	Wang *et al.*^45^
Graphene	0.1 mg mL^−1^	Alginate hydrogels	Ball-on-disc, 15 N	0.106	Wu *et al.*^46^
Graphene + MoS_2_	0.5%	Polyalkylene glycol (PAG)	Ball-on-disk, 75 N	∼0.08	Gong *et al.*^47^
Multi-walled carbon nanotubes (MWCNTs) & titanium dioxide (TiO_2_)	MWCNTs-0.1 wt%	Turbine meter oil	Designed device, 7 N	MWCNTs ∼ 1.43	Pourpasha *et al.*^48^
	TiO_2_-0.05 wt%			TiO_2_ ∼ 1.39	
CDs@polydiallyl dimethylammonium chloride (PDDA)	3 wt%	PEG-200	Ball on disk, 5 N	0.079	Chen *et al.*^49^
CQDs	0.15 wt%	Pongamia oil	Pin on disk, 133 N	0.084	This work

As shown in Table 3, the CQD-modified Pongamia oil exhibited a COF of 0.084 at an optimum concentration of 0.1 wt% under a load of 133.33 N. Although direct comparison is limited by differences in base oils, additive concentrations, contact configurations, loads, and operating conditions, the obtained COF is comparable to those reported for other carbon-based lubricant additives. These results demonstrate the potential of cowpea-derived CQDs as effective low-concentration additives for improving the lubricating performance of Pongamia oil.

### 3.7. Thermal conductivity (*k*), flash and fire points

Thermal conductivity is an intrinsic thermophysical property of the oil that quantifies its ability to conduct heat *via* molecular or atomic interaction. Lower thermal conductivity implies that the oil will resist heat flow and act as a thermal insulator, which results in oxidative degradation and increased wear and friction. Whereas higher thermal conductivity denotes an efficient transfer of heat, and a reduction in the temperature within the system, leading to reduced heating of the machinery due to friction between two closely moving surfaces.^50,51^

**Fig. 10:**
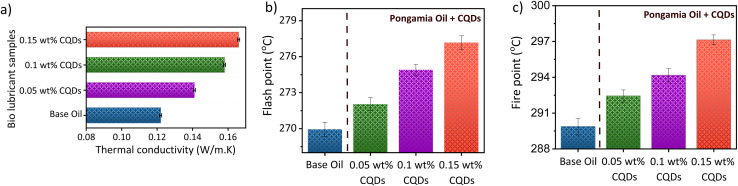
(a) Thermal conductivity of base Pongamia oil and CQDs modified Pongamia oils (0.05–0.15 wt%). (b) Flash point of base Pongamia oil and CQDs modified Pongamia oils (0.05–0.15 wt%). (c) Fire point of base Pongamia oil and CQDs modified Pongamia oils (0.05–0.15 wt%).

The bar graph of thermal conductivity presented in Fig. 10a reveals a systematic, concentration-dependent enhancement of Pongamia oil upon the incorporation of CQDs. The Pongamia oil exhibited thermal conductivity of 0.122 ± 0.0005 W m^−1^ K^−1^ (Table 4). The lower thermal conductivity is due to the presence of rich triglycerides that are polymeric in nature. On addition of 0.15% of CQDs, the overall thermal conductivity was increased to up to 0.166 ± 0.0005 W m^−1^ K^−1^. Thereby, a relative increase of 36.0% in base Pongamia oil is observed after the addition of CQDs.

**Table 4:** The thermal conductivity and relative increase of base Pongamia oil and CQDs modified Pongamia oils (0.05–0.15 wt%)

Sample	Thermal conductivity (W m^−1^ K^−1^)	Relative increase (%)
Base Pongamia oil	0.122 ± 0.0005	—
Pongamia oil + 0.05 wt% CQDs	0.141 ± 0.0005	15.57
Pongamia oil + 0.1 wt% CQDs	0.158 ± 0.0005	29.5
Pongamia oil + 0.15 wt% CQDs	0.166 ± 0.0005	36.06

This enhancement in thermal conductivity can be attributed to the unique physicochemical properties of CQDs, including their oxygen-containing functional group, high surface area and better colloidal stability. These positive effects highlight the potential of CQDs to be used as a nano-additive in bio-lubricant matrices.

The flash and fire points are the two parameters to evaluate the thermal safety of Pongamia oil. Flash point is the minimum temperature at which sufficient vapours exist so that they will ignite momentarily. The fire point is the minimum temperature at which the vapours will burn continuously for five seconds. Higher flash and fire points correlate to improved oxidation stability and reduced volatility.^42^

The thermal analysis demonstrated in Fig. 10b and c exhibits an increase in thermal performance after the addition of CQDs to base Pongamia oil. The flash and fire points of base Pongamia oil were exhibited at 270 ± 0.6 °C and 289 ± 0.3 °C, respectively (Table 5). However, after the addition of CQDs, the flash and fire points elevated to 277 ± 0.5 °C and 297 ± 0.4 °C, respectively.

**Table 5:** The flash and fire points of base Pongamia oil and CQDs modified Pongamia oils (0.05–0.15 wt%)

Sample	Flash point (°C)	Fire point (°C)
Base oil	270 ± 0.6	289 ± 0.3
0.05 wt% CQDs	272 ± 0.5	292 ± 0.5
0.10 wt% CQDs	274 ± 0.4	294 ± 0.5
0.15 wt% CQDs	277 ± 0.5	297 ± 0.4

The observed enhancements can be attributed to several synergetic effects. First, the abundant oxygen functional group present on the surface of the CQDs creates hydrogen bonding and van der Waals interactions with triglycerides of the Pongamia oil. Second, the ability of the CQDs act as nanoscale thermal barriers by effectively redistributing the heat generated and thereby delaying the onset of ignition. Finally, the addition of CQDs increases the viscosity of base Pongamia oil, thereby reducing volatility and promoting film persistence. Consequently, a larger amount of energy is needed to reach the vapor concentration at which ignition will occur. This is also consistent with the experimental observation of elevated flash and fire points.

### 3.8. Mechanistic insight into the reduction of wear and COF

**Fig. 11:**
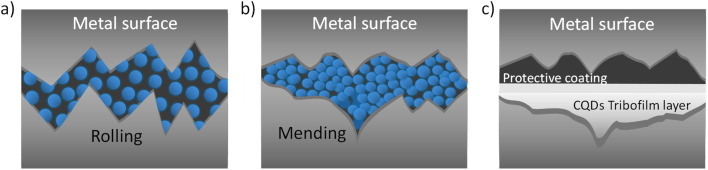
(a) Rolling mechanism of the CQDs between the metal surfaces. (b) Mending mechanism of the metal surfaces. (c) Formation of a CQDs tribofilm layer between two metal surfaces.

The reduction of friction and wear on addition of CQDs can typically be explained *via* three possible mechanisms as depicted in Fig. 11. A smaller particle size of CQDs (7.06 ± 1.8 nm) allows them to act as rolling elements within the sliding interface. This can partially convert sliding motion into rolling motion, thereby decreasing the COF.

In addition, CQDs can also lead to polishing and mending of the metallic surface during the sliding motion. The nano size and spherical morphology (Fig. 2) of CQDs enable them to penetrate and deposit within micro grooves and defects. This filling of CQDs smoothens the worn surfaces, enhancing surface uniformity. Thereby, resulting in reduced wear (Fig. 7b).

Relative reduction of COF and wear on the addition of CQDs to Pongamia oil can also be explained *via* the mechanism of possible tribofilm formation. Due to their nanoscale quantum size and higher surface energy, CQDs can possibly form a tribofilm under applied shear rate, pressure and temperature. The possibly formed tribofilm acts as a protective barrier that separates two metallic surfaces, reducing direct contact between them and mitigating the surface asperities.

## 4. Conclusion

Unlike the usually employed synthetic oil and metal-based nanoparticles, causing environmental harm. This work revealed the use of Pongamia oil as a lubricant and CQDs synthesized from cowpea seeds as nanoadditives. At the concentration of 0.15 wt% CQDs, the Pongamia oil increased the viscosity, thereby contributing to the separation of two metal surfaces. Besides this improvement, addition of CQDs further reduced COF, wear, and increased thermal conductivity, flash, and fire points. The enhanced tribological performance of CQDs induced Pongamia oil could be attributed to the various synergetic mechanisms of the CQDs nanoadditives. The wear surface analyzed exhibited fewer wear scars and grooves in the CQDs induced Pongamia oil metal, suggesting that the role of oxygen presence on the surface of CQDs. The EDS is composed of carbon and oxygen, robust enough to protect the metallic surface, possibly leading to the development of a carbon-rich protective layer. The results provide alternate and effective use of bio-based oils with CQDs as nanoadditives to enhance tribological applications.

## Author contributions

The manuscript was written through contributions of all authors. All authors have given approval to the final version of the manuscript.

## Conflicts of interest

There are no conflicts to declare.

## Supplementary Material

RA-OLF-D6RA03760G-s001

## Data Availability

The data supporting this article have been included as part of the manuscript and the supplementary information (SI). Supplementary information: detailed materials and methods of Pongamia oil analysis and data relevant to zeta potential, flow curve and EDS analysis of the metal. See DOI: https://doi.org/10.1039/d6ra03760g.
