# Supplementary material for: Green carbon quantum dots as eco-friendly nano-additives for tribological enhancement of Pongamia oil
Source: RSC Adv. 2026 Aug 4;16(39):44585–96. doi: 10.1039/d6ra03760g (PMC13435042; doi:10.1039/d6ra03760g)
Supplement: Supplementary file 1 [file RA-016-D6RA03760G-s001.pdf]

Supplementary Information (SI) for RSC Advances.
This journal is © The Royal Society of Chemistry 2026

Green carbon quantum dots as eco-friendly nano-additives for tribological enhancement of Pongamia oil

Mohammed Ali Khan[1], Akshatha R Shetty[1], Ganesha Aroor [1], Meghana K Navada [1*]

[1] Manipal Institute of Technology, Manipal Academy of Higher Education, Manipal, India.

*Corresponding author (meghana.navada@manipal.edu)

1. Materials and methods

1.1. Physicochemical Characterisation of Pongamia Oil

1.1.1. Acid value

The 10g of oil sample was weighed, and 60 mL of neutralized ethyl alcohol (Sigma Aldrich) was transferred into a 250 mL conical flask, and phenolphthalein indicator was added to the conical flask. Then, the sample mixture was heated for 15 minutes, and the hot solution was titrated against 0.1N KOH until the colorless solution turned into a permanent light pink color [1].

1.1.2. Iodine value

The 0.3g of oil and 20 mL of carbon tetrachloride (Sigma Aldrich) were taken in a 250 mL flask. The blank was prepared with only 20 mL of carbon tetrachloride. Then pipetted 25.0 mL of Wijs reagent (Iodine monochloride) was added to each flask. The flask was stoppered and kept in the dark for 30 min. Then 10 mL of 30% potassium iodide (KI) was added and mixed well. Then immediately titrated against 0.1N sodium thiosulphate (Sigma Aldrich) until the yellow color disappeared. Then 1 mL of the starch indicator was added and titrated with continuous stirring until the blue color of the starch indicator disappeared (endpoint)[1].

1.1.3. Saponification value

The 2g of oil and 0.5 N alcoholic KOH were taken in the conical flask, and the solution mixture was refluxed in the water bath for 45 mins. Then cool the solution mixture and add 2-3 drops of phenolphthalein indicator and titrate against 0.5N HCl taken in the burette. The endpoint is until the pink color disappears completely[1].

1.1.4. Peroxide value

The 2g of oil and 30 mL of chloroform (Sigma Aldrich) were taken in a 250 mL flask. The blank was prepared with only 30 mL of chloroform. Then, 5% of 0.5 mL KI was put into each flask. The flask was stopped and kept in the dark for 10 min. Then 20 mL of distilled water was added and mixed well. Then immediately titrated against 0.1N sodium thiosulphate until

the yellow color disappeared. Then 1 mL of the starch indicator was added and titrated with continuous stirring until the blue color of the starch indicator disappeared (endpoint)[1].

1.1.5. Determination of oil density

The density of the oil was measured using a specific gravity bottle.

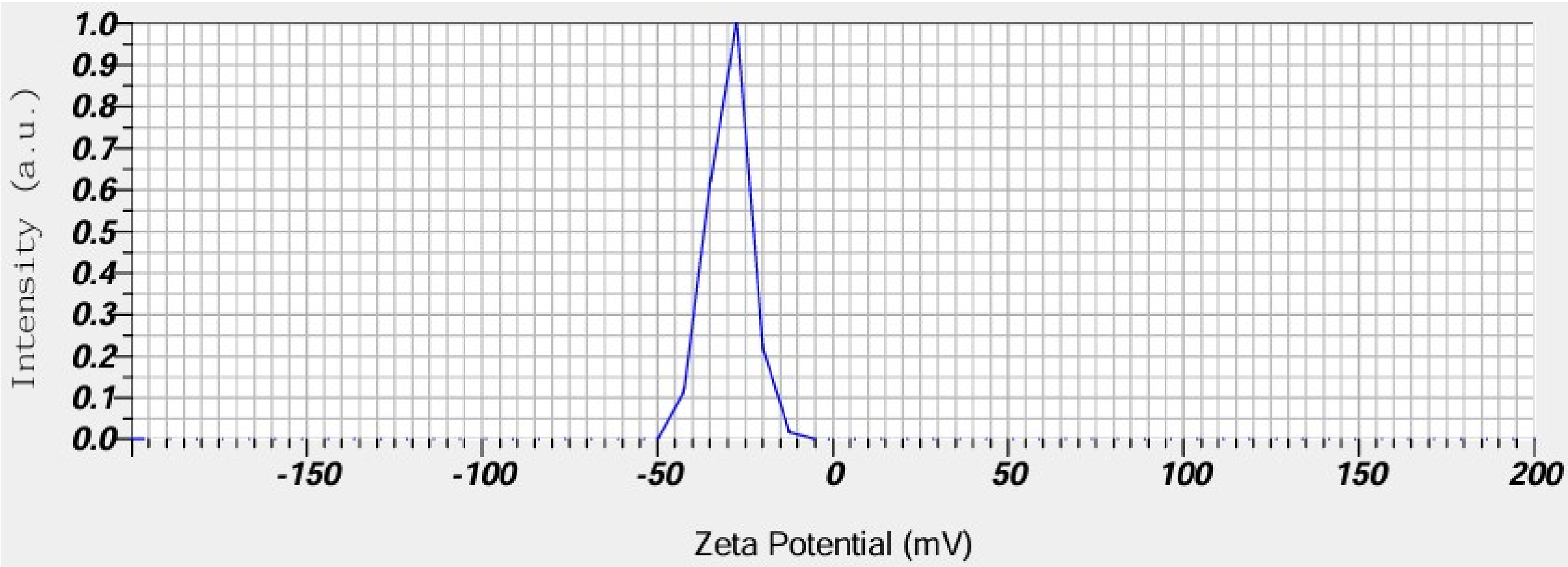

Figure S1. Zeta potential of the as-synthesized CQDs.

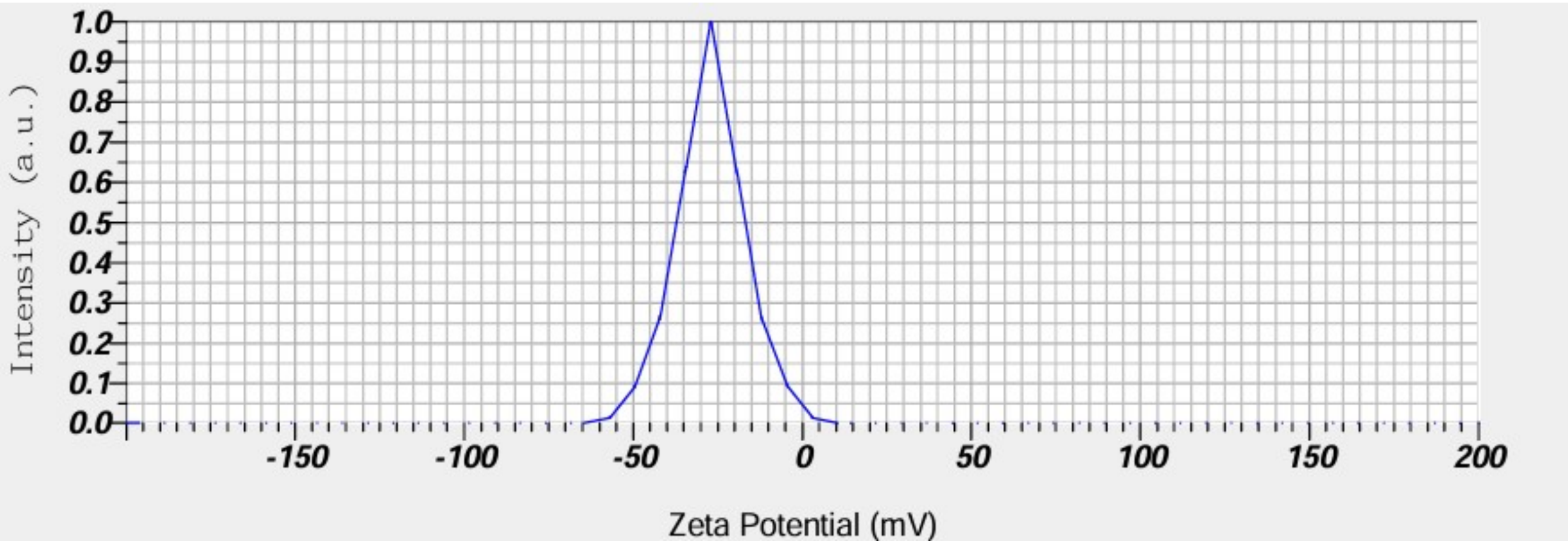

Figure S2. Zeta potential of the 0.15 wt% CQDs modified Pongamia oil.

Table S1. The activation energy and R^2 fitting values using Arrhenius model

Base oil		0.05 wt% CQDs + base oil		0.1 wt% CQDs + base oil		0.15 wt% CQDs + base oil	
Activation energy (J/mol)	R^2	Activation energy (J/mol)	R^2	Activation energy (J/mol)	R^2	Activation energy (J/mol)	R^2
28910	0.997	29292.36	0.997	0.995	0.998	30279.5	0.997

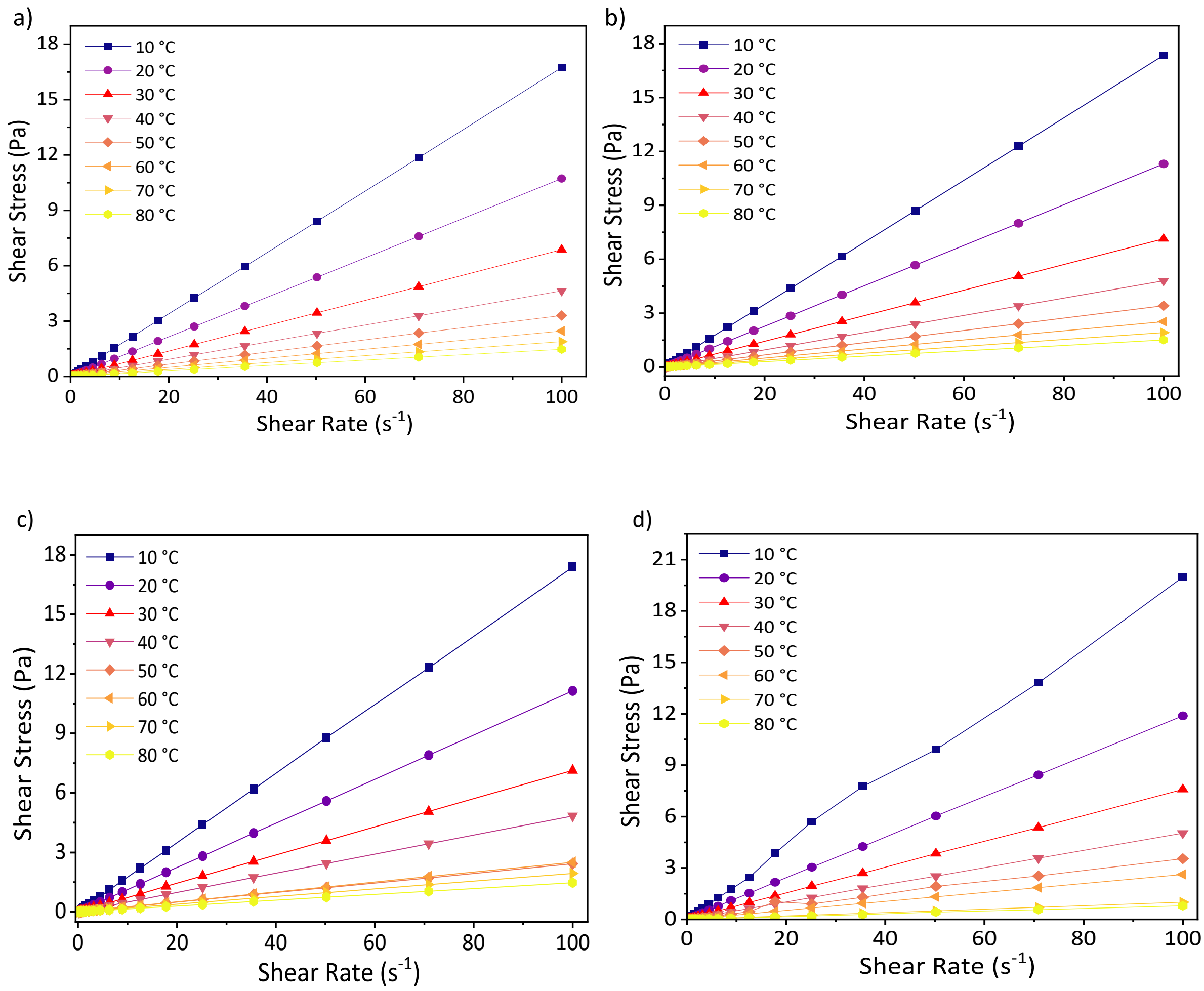

Figure S3. Temperature-dependent flow behaviour of the fluids. a) Flow curve of pristine Pongamia oil b) Flow curve of 0.05% CQDs modified Pongamia oil. c) Flow curve of 0.1% CQDs modified Pongamia oil. d) Flow curve of 0.15% CQDs modified Pongamia oil.

Table S2. The flow behavior index and R^2 fitting values using Herschel-Bulkley model

	Base oil		0.05 wt% CQDs + base oil		0.1 wt% CQDs + base oil		0.15 wt% CQDs + base oil	
Temperature (°C)	Flow behavior index (n)	R^2	Flow behavior index (n)	R^2	Flow behavior index (n)	R^2	Flow behavior index (n)	R^2
10	0.993	0.999	0.997	0.999	0.995	0.999	0.9626	0.998
20	1.000	1	0.998	1	0.995	1	0.9985	0.999
30	0.997	0.999	0.996	1	0.993	1	0.9927	0.999
40	0.998	1	1.002	1	0.992	1	0.9936	0.999
50	1.000	0.999	1.002	1	0.982	0.999	0.917	0.995
60	0.996	0.999	1.000	0.999	0.991	0.999	1.001	0.999
70	1.002	0.999	0.997	0.999	0.993	0.999	1.002	0.999
80	0.991	0.999	1.000	0.999	0.993	0.999	1.004	0.999

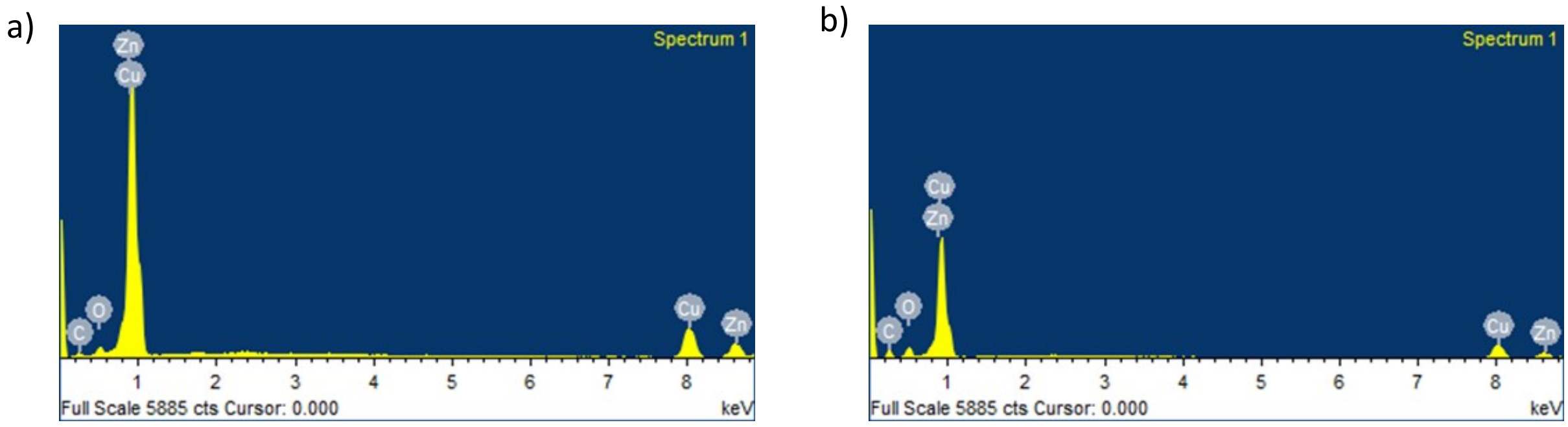

Figure S4. a) EDS spectrum of the metal without lubricant b) EDS spectrum of the metal with CQDs induced Pongamia oil.

References

(1) Kashinath, M.; Gupta, A. R.; Krishnan, Dr. K.; Sakpal, V. A.; Sharma, S. V. Physicochemical Evaluation of Used Frying Oils Through Determination of Saponification, Acid, Peroxide, And Iodine Values. *International Journal of Current Science Research and Review* **2025**, *08* (05). https://doi.org/10.47191/ijcsrr/V8-i5-15.
